# Review of Current Immunologic Therapies for Hidradenitis Suppurativa

**DOI:** 10.1155/2017/8018192

**Published:** 2017-08-20

**Authors:** Victoria K. Shanmugam, Nadia Meher Zaman, Sean McNish, Faye N. Hant

**Affiliations:** ^1^Division of Rheumatology, Ideas to Health Laboratory, The George Washington University, School of Medicine and Health Sciences, 701 Ross Hall, 2300 Eye Street, NW, Washington, DC 20037, USA; ^2^Division of Rheumatology and Immunology, Medical University of South Carolina, 96 Jonathan Lucas Street, Suite 822, Charleston, SC 29425, USA

## Abstract

Hidradenitis suppurativa (HS) is a chronic, recurrent, inflammatory disease of apocrine gland-bearing skin which affects approximately 1–4% of the population. The disease is more common in women and patients of African American descent and approximately one-third of patients report a family history. Obesity and smoking are known risk factors, but associations with other immune disorders, especially inflammatory bowel disease, are also recognized. The pathogenesis of HS is poorly understood and host innate or adaptive immune response, defective keratinocyte function, and the microbial environment in the hair follicle and apocrine gland have all been postulated to play a role in disease activity. While surgical interventions can be helpful to reduce disease burden, there is a high recurrence rate. Increasingly, data supports targeted immune therapy for HS, and longitudinal studies suggest benefit from these agents, both when used alone and as an adjunct to surgical treatments. The purpose of this review is to outline the current data supporting use of targeted immune therapy in HS management.

## 1. Introduction

Hidradenitis suppurativa (HS) is a chronic, recurrent, inflammatory disease of the apocrine sweat glands, characterized by recurrent abscessing inflammation [1]. The prevalence of HS is estimated at 1–4% in young adults [2–5]. Women are affected more commonly than men (with a female to male ratio of 3 : 1), and the disease is more common in African Americans (AA) [6]. Patients with HS develop inflammatory nodules, abscesses, and sinus tracts around the apocrine glands, and increasing evidence points to an immune basis for this disease [7].

The Hurley staging system (Table 1) is a three-stage classification system developed for assessing extent and severity of HS, and in particular for identifying patients who need surgical intervention [1, 8]. Until now, surgery has been a mainstay of HS management, and surgery remains the first-line therapy for patients with extensive Hurley stage III disease [9]. Studies show that, in severe disease, the best results are achieved with wide local excision [10–12], but even with extensive surgical intervention HS often recurs and this has led to the recent interest in the use of adjunctive biologic therapy in the management of HS [13–15].

While several risk factors including obesity and smoking are known to be associated with disease activity in HS, the molecular drivers of HS are unknown and are actively being investigated [16]. Host innate or adaptive immune response [7], defective keratinocyte function [1], and the microbial environment in the hair follicle and apocrine gland [17] have all been postulated to play a role in disease activity. Recent studies suggest that tumor necrosis factor *α* (TNF*α*) is elevated in active lesions. In addition, interferon-gamma (IFN-*γ*) has been shown to be elevated in lesion effluent [18], and suppression of IL22 [19] along with the IL12/23 pathways is thought to play a role in pathogenesis [20, 21].

The purpose of the current manuscript is to review the data supporting the use of the currently available immunologic therapies for HS and to provide an update on novel immune mechanisms under investigation in HS.

## 2. Currently Available Immune Therapies

### 2.1. Antibiotics

Antibiotics have been advocated as first-line therapy for HS for many years [1]. However, routine cultures of HS lesions often fail to identify infection or identify only normal skin flora [22].

Recent use of next-generation sequencing techniques using 16S and 18S ribosomal RNA to identify microbial profiles of HS lesions has shown that there are differences between lesional and nonlesional HS skin [22], with the microbiome of lesional skin often demonstrating* Corynebacterium* species and* Porphyromonas* and* Peptoniphilus* species.* Porphyromonas* and* Peptoniphilus* species were not detected in healthy controls. In contrast, healthy skin showed a relatively higher abundance of* Propionibacterium* species, indicating that dysbiosis of the cutaneous microbiome may play a role in HS [22].

Clinically it is acknowledged that while antibiotics play a role in the management of HS [23, 24], their efficacy is often short-lived. Antibiotic resistance, along with complications from chronic antibiotic use, is a concern. In parallel with investigations targeting immune pathways in HS, the interplay of the immune response with the microbiome should be considered. In particular biofilm formation, which is known to be associated with delayed healing in chronic wounds [25, 26], may play a role in advanced disease. Biofilms have been shown to be present in established HS lesions [27], although they are less prominent in early lesions. Further investigation into biofilms in HS, and in particular antimicrobial peptide pathways that assist with microbial clearance, may identify novel therapeutic pathways in the management of HS [28–31].

### 2.2. Steroid Therapy

Glucocorticoids are naturally occurring hormones that have been used since the 1950s as anti-inflammatory therapy for immune mediated diseases [32]. Glucocorticoid receptors are present in the cytoplasm of almost all human cells. Upon binding to the glucocorticoid domain the receptor translocates to the nucleus and binds to glucocorticoid response elements in DNA, resulting in transcription of steroid responsive genes. Glucocorticoids have very wide-ranging clinical effects due to their potential to increase transcription of a variety of cytokines, chemokines, and inflammatory adhesion molecules [33, 34].

In the management of HS, intralesional steroid therapy is often used to treat isolated nodules (e.g., triamcinolone (3–5 mg) injected into the nodules); however, there is minimal data to support its use in patients with extensive disease [24, 35]. Similarly, while systemic corticosteroids in HS are often advocated, there are no randomized controlled trials to support their use, and due to the wide-ranging actions of steroids, side effects limit their role in HS [24]. In a study investigating low dose systemic prednisone as an adjunct to other therapies there was some benefit, but it was largely seen in the patients who received steroids in conjunction with anti-TNF-*α* agents [36].

### 2.3. Therapies Blocking Tumor Necrosis Factor-*α*

TNF-*α* is a central cytokine that regulates immune responses in several inflammatory diseases.

Agents blocking TNF-*α* have been marketed for many years for the treatment of rheumatoid arthritis, Crohn's disease, and other inflammatory diseases. Several anti-TNF-*α* agents have been utilized in the treatment of HS. Infliximab (Remicade®) is a chimeric monoclonal antibody that binds soluble and cell-bound TNF-*α* and thus inhibits the actions of circulating TNF-*α* while also inducing apoptosis in cells with TNF-*α* bound to their receptor. Adalimumab (Humira®) is a fully human monoclonal antibody which binds the soluble and transmembrane forms of TNF-*α*. Etanercept (Enbrel®) is a fusion protein created by linking the extracellular binding regions from two TNF-RII (p75) receptors to the FC portion of human IgG1. It binds both soluble TNF-*α* and lymphotoxin (TNF-*β*).

Early trials of TNF-*α* inhibitors in HS were limited due to challenges defining clinical outcome measures. Etanercept was shown to be ineffective for HS [37]. However, a small trial of infliximab therapy (*n* = 38) showed a 50% reduction in baseline HS Severity Index Score in the treated group compared to placebo along with statistically significant improvements in Dermatology Quality of Life Index (DQLI), and inflammatory markers [38]. Infliximab has never been through rigorous phase II and phase III studies for HS and thus does not have regulatory approval for HS. However, the results of this and other early studies [39, 40] suggest that infliximab is effective in HS [41]. Based on data from pharmacokinetic studies, the addition of methotrexate to infliximab increases patient exposure to infliximab by approximately 30%, and thus many rheumatologists use these agents in combination to increase the efficacy from infliximab particularly in the HS patients who have concurrent inflammatory arthritis.

Adalimumab has been more thoroughly studied in phase II and two phase III clinical trials. Using the novel endpoint of the Hidradenitis Suppurativa Clinical Response (HiSCR) defined as a 50% reduction in active nodule (AN) count without increase in abscesses or draining fistulae, the PIONEER I and II studies were able to demonstrate efficacy of adalimumab at a dose of 40 mg weekly subcutaneously for HS. It should be noted that these studies were conducted in a predominantly Caucasian population, excluded patients with more severe Hurley stage III disease, and also excluded patients undergoing surgery or receiving concurrent opiate analgesia. Longitudinal studies investigating observational cohorts of HS patients that are more representative of the HS population seen in the United States are ongoing.

## 3. Novel Immune Pathways and Therapies Being Investigated for HS

### 3.1. Phosphodiesterase 4 (PDE4)

Apremilast (Otezla®) is a small molecule selective inhibitor of phosphodiesterase 4 (PDE4). Inhibition of PDE4 impacts cellular cyclic AMP production and thus reduces TNF-*α* production and secondarily reduces IL17 and IL23 while increasing IL10. Developed and FDA approved as an oral agent specifically for use in psoriasis and psoriatic arthritis, this agent has been utilized in small case series of patients with HS with some efficacy [42].

### 3.2. IL12/23 Pathways

The IL12/23 pathway is implicated in HS based on small studies demonstrating expression of IL12/23 in macrophages infiltrating HS lesional skin [43] and infiltration of the HS dermis by IL17 producing T-helper cells. Additionally, single nucleotide polymorphisms in the IL12 receptor beta-1 impact the clinical phenotypes of HS [44]. Ustekinumab (Stelara®) is a human monoclonal antibody which targets the common p40 subunit of IL12 and IL23, preventing receptor binding and T cell activation. Ustekinumab is FDA approved for psoriasis, psoriatic arthritis, and Crohn's disease. Case reports have demonstrated some benefit of ustekinumab in HS [45–47].

### 3.3. IL-1 Pathways

Anakinra (Kineret®) is a recombinant IL-1 receptor antagonist which inhibits the biologic activity of IL-1 by competitively binding to the IL-1 receptor [48]. This agent was originally developed for the treatment of rheumatoid arthritis but has more recently been adopted for use in autoinflammatory syndromes including familial Mediterranean fever and adult onset Still's disease. Use of IL-1 receptor antagonist therapies in HS have had mixed results with some studies showing lack of efficacy [49, 50], but small case series and a recent randomized controlled trial have shown efficacy in moderate to severe HS [51, 52]. In this double-blind randomized trial, anakinra efficacy correlates with alterations in cytokine production by PBMC, with increased production of IFN-*γ* in the placebo arm and increased IL-22 in the anakinra treated group. These observations correlate with studies investigating cytokine production in HS lesion tissue and effluent in which IFN-*γ* has been shown to be elevated in active disease [18], and IL-22 production is reduced [19], providing further evidence that targeting biologic response pathways in HS can provide novel therapeutic options for this disease.

### 3.4. Complement Pathways

Another pathway that has generated interest as a potential target for novel HS therapy is the complement pathway, which has been shown to be active in patients with HS [53]. An open label phase II study is currently underway in Greece to investigate the safety of the complement inhibitor IFX-1 in patients with moderate to severe HS (NCT 03001622). The results of this study are eagerly awaited, given its potential to add to the therapeutic armamentarium in HS.

### 3.5. Antimicrobial Peptide Production

Abnormalities in keratinocyte antimicrobial peptide (AMP) production may also contribute to defective keratinocyte function and impaired microbial clearance seen in HS [28–31]. These observations are further supported by the data indicating that IL-22 production is reduced in active disease and increases in response to therapy [54]. In vitro data investigating keratinocyte function in HS has corroborated this finding, demonstrating reduced IL-22 release from keratinocytes harvested from HS lesions compared to those from healthy skin. IL-22 is a member of the IL-10 family of cytokines which play a role in cutaneous innate immunity and stimulate antimicrobial peptide (AMP) production. As the role of AMP in HS and cytokines driving AMP production are further elucidated, agents targeting the AMP pathways may represent novel therapies for this disease.

## 4. Recommendations on Timing of Immune Therapies Relative to Surgical Interventions in HS

Patients with severe Hurley stage III disease are often excluded from clinical trials of biologic agents because of concerns that initiating immunosuppressive therapy without surgical intervention carries the risk of disseminating infection. Studies show that wide local excision surgery is often the most effective way to rapidly improve the quality of life for patients with Hurley stage III disease [10]. However, a series of recent studies suggest that patients treated with adjuvant biologic therapy after radical resection of HS demonstrated lower rates of recurrence and disease progression, as well as longer disease-free interval [13]. Data from the Wound Etiology and Healing-Hidradenitis Suppurativa (WE-HEAL-HS) study has shown that combining biologic immunosuppression therapy with wide local excision surgeries is more effective than either intervention alone, and data suggests that timing of biologic therapy relative to surgery does not alter the synergistic impact of these interventions [55].

## 5. Long Term Impact of Uncontrolled Inflammation in HS

Population based studies show that HS is associated with a significantly increased risk of adverse cardiovascular outcomes and all-cause mortality independent of confounders [56]. It has been postulated that, similar to the association seen in rheumatoid arthritis and other chronic inflammatory conditions, the increased risk of cardiovascular disease may be explained by uncontrolled inflammation. These observations further support the need for identifying more effective therapies for HS and for longitudinal outcomes studies in HS to investigate the impact of immune suppression in this patient population.

## 6. Conclusions

The role of the immune system in driving HS is increasingly recognized and various therapies are under investigation for efficacy as treatment for HS. Based on data from randomized controlled clinical trials, the TNF*α* inhibitor adalimumab recently received orphan drug designation for HS. As new immune pathways driving disease pathogenesis are recognized, there is a significant need for ongoing clinical studies investigating adjunctive immune therapies in HS.

## Figures and Tables

**Table 1:** Hurley staging system.

Stage	
0	No active HS
I	Localized abscess, no sinus tracts
II	Recurrent abscesses with sinus tracts and scarring, single or multiple widely separated lesions
III	Diffuse involvement with multiple interconnected sinus tracts and abscesses

## Abbreviations

HS: Hidradenitis suppurativa
IL: Interleukin
IFN-*γ*: Interferon-gamma
TNF-*α*: Tumor necrosis factor-alpha.
